# Supplementary material for: Opportunities for Tailored Support to Implement Smoke-Free Homes: A Qualitative Study among Lower Socioeconomic Status Parents
Source: Int J Environ Res Public Health. 2019 Dec 27;17(1):222. doi: 10.3390/ijerph17010222 (PMC6981932; doi:10.3390/ijerph17010222)
Supplement: Supplementary file 1 [file ijerph-17-00222-s001.pdf]

Table S1. Factors in the implementation of home smoking rules (HSRs) reported by parents with a complete smoke-free home (SFH).

Respondent number and parental smoking status	R09 Non-smoker Single parent	R01 Non-smoker Partner: non-smoker	R05 Non-smoker Partner: smoker	R14 Non-smoker Partner: smoker	R10 Smoker Partner: single parent
**Initiator of HSRs**	Respondent	Respondent	Respondent	Respondent and partner	Respondent
**Introduction of HSRs**	When child was born	During pregnancy	When started own household<br>When started living together	When moved house<br>When children were born	When painted her house
**Content of HSRs**	No smoking indoors<br>Smoking at balcony	No smoking indoors<br>Smoking in front of house<br>Smoking in garden	No smoking indoors<br>No in car with children<br>Smoking in garden	No smoking indoors<br>No smoking near children<br>Smoking in back of garden<br>Smoking in shed	No smoking indoors<br>Smoking in garden<br>Smoking in shed
**Motivation for HSRs**	Healthy environment for child to grow up in	Health of children<br>Smoke is dirty	Healthy growing up of children<br>Being good example: prevent children from becoming a smoker<br>Smoke is dirty	Health of children<br>Smoke is dirty<br>Clean and tidy garden	Children find smoke unpleasant<br>Keep house fresh and neat
**Acceptation of HSRs**	Rules are accepted<br>Few smokers in social environment<br>Visitors smoke automatically outside	Rules are accepted<br>Initially resistance from family members<br>Visitors smoke automatically outside without complaints	Rules are accepted<br>Initially broken contact with friends and family member<br>Visitors smoke automatically outside	Rules are accepted<br>Partner and visitors smoke automatically outside	Rules are accepted<br>More and more people smoke outside<br>Children satisfied with rules
**Enforcement of HSRs**		No exceptions or violations allowed (assertiveness)<br>Driven to maintain rules<br>Difficult at first, later easy to maintain rules	No exceptions or violations allowed (assertiveness)<br>Driven to maintain rules<br>Easy to maintain rules	No exceptions or violations allowed indoors<br>Easy to maintain indoor rules<br>Outdoor rules more flexible (only for smoking visitors)	No exception or violations allowed (willpower)<br>Clear rules and clear communication about rules<br>Easy to maintain rules<br>Giving good example herself
**Smoking habits and HSRs**	When still smoking, already used to smoke outdoors				At first difficult to change smoking habit from indoors to outdoors<br>Outdoor smoking is habit now
**Barriers to SFH**				Previously: bad weather	Previously: bad weather

Respondent number and parental smoking status	R09 Non-smoker Single parent	R01 Non-smoker Partner: non-smoker	R05 Non-smoker Partner: smoker	R14 Non-smoker Partner: smoker	R10 Smoker Partner: single parent
**Facilitators of SFH**		Smoking of partner<br>Child birth increased acceptance of rules		Home without proper ventilation opportunities<br>Scullery (previously the separate smoking room) became part of the home	No other smokers in the home<br>Nowadays visitors ask where they may smoke
**Extra advantages of SFH**	Could have served as step towards smoking cessation		Healthier environment (indoors and outdoors)<br>Could be step towards smoking cessation partner	Fresh and healthier house<br>Could be step towards smoking cessation partner	Fresh house<br>Contributes to smoking less<br>Contributes to quit attempts
**Role of children in SFH**			Parents could set rules together with children<br>Children could expose parents to rules		Children could exert pressure on parents

Table S2. Factors in the implementation of home smoking rules (HSRs) reported by parents with flexible smoke-free home (SFH).

Respondent number and parental smoking status	R11 Smoker Partner: non-smoker	R08 Smoker Partner: smoker	R12 Smoker Partner: smoker
**Initiator of HSRs**	Respondent	Respondent and partner	Respondent and partner
**Introduction of HSRs**	When child was born	When child was born<br>When door between kitchen and living room was removed	During pregnancy
**Content of HSRs**	No smoking indoors<br>Smoking in garden	No smoking indoors<br>Smoking in garden	No smoking indoors<br>No smoking in car<br>Smoking in garden
**Motivation for HSRs**	Feeling guilty: children don't ask to live in smoke<br>Health of everyone<br>Dirty house	Not wanting to expose children to smoke<br>Feeling guilty: children may blame her for becoming ill later in life<br>Better for everyone	Not wanting to expose children to smoke<br>Feeling guilty: being responsible for child's health<br>Dirty house
**Acceptation of HSRs**	Rules are accepted<br>Most people they know have children and smoke outdoors	Rules are accepted<br>Most people they know have children and smoke outdoors	Rules are accepted<br>It was always a family rule to smoke outdoors
**Enforcement of HSRs**	No exceptions allowed<br>Sometimes violations (e.g., lighting up indoors, shortly entering the house while smoking)<br>Easy to maintain rules	Sometimes exceptions (e.g., smoking inside when children are asleep, smoking inside during parties in case of bad weather)<br>Rules rather unclear, exceptions not discussed	Mostly no exceptions or violations of rules<br>Sometimes exceptions (e.g., smoking at the front door when it is dark and bad weather outside)<br>Easy to maintain rules (normal and accepted)

Respondent number and parental smoking status	R11 Smoker<br>Partner: non-smoker	R08 Smoker<br>Partner: smoker	R12 Smoker<br>Partner: smoker
**Smoking habits and HSRs**	Habit has changed to smoking outdoors<br>Habit more important than smoking itself	At first difficult to change smoking habit from indoors to outdoors<br>Gradually replaced smoking indoors by smoking outdoors<br>Smoking (outdoors) has become less important	At first difficult to change smoking habit from indoors to outdoors<br>Now smoking outdoor has become the habit
**Barriers to SFH**	Bad weather		Nicotine addiction<br>Bad weather
**Facilitators of SFH**	Norm in social environment is smoking outdoors<br>Having a comfortable outdoor place to smoke	Nice weather<br>No separate room for smoking available<br>Shelter available to smoke outside	Farewell ritual (e.g., celebrating last time smoking indoors; cleaning house once smoking outdoors)<br>Removing ashtrays from inside the house
**Extra advantages of SFH**	Fresh house<br>Less smoking, although no intention to quit	Fresh house and less cleaning/ventilating<br>Lower heating costs<br>Less smoking, with the intention to quit<br>May contribute to smoking cessation	Fresh house<br>Healthy child<br>Less smoking, with the intention to quit<br>Step towards smoking cessation
**Role of children in SFH**	Could remind parents about HSRs<br>Determining a reward for obeying rules	Could support parents in HSRs/SFH<br>Could remind parents about rules<br>Could support parents in smoking cessation	Could support parents in HSRs/SFH

Table S3. Factors in the implementation of home smoking rules (HSRs) reported by parents with a partial smoke-free home (SFH).

Respondent number and parental smoking status	R04 Non-smoker<br>Single parent	R07 Non-smoker<br>Partner: smoker	R02 Smoker<br>Single parent	R03 Smoker<br>Partner: non-smoker	R06 Smoker<br>Partner: non-smoker	R13 Smoker<br>Partner: smoker
**Initiator of HSRs**	Respondent	Respondent and partner	Respondent	Respondent and partner	Partner	Respondent and partner
**Introduction of HSRs**	Unclear rules are changing continuously	When started living together	When child was born		One year ago	When child was born

Respondent number and parental smoking status	R04 Non-smoker Single parent	R07 Non-smoker Partner: smoker	R02 Smoker Single parent	R03 Smoker Partner: non-smoker	R06 Smoker Partner: non-smoker	R13 Smoker Partner: smoker
**Content of HSRs**	No clear rules<br>Preferred rule: no smoking indoors<br>Difficulties with indicating and setting rules	Smoking only allowed in the kitchen<br>No smoking near children	Smoking only allowed in the kitchen	Smoking only allowed in the kitchen and only when ventilating<br>No smoking near children	Smoking only allowed in the kitchen and only when ventilating	Smoking only allowed in the kitchen, only when ventilating<br>Smoking indoors not allowed during the day<br>No smoking near children<br>Rule in principle: smoking in garden
**Motivation of HSRs**	Being a non-smoker<br>Smoke is dirty	Health of children<br>Smoke is dirty<br>Being good example: prevent children from becoming a smoker	Feeling guilty for smoking in presence of children	Children	Puppies<br>Partner finds smoke annoying and dirty	Children<br>New house
**Acceptation of HSRs**	Rules not accepted<br>No communication about rules<br>Family members and visitors smoke indoors	Rules accepted<br>Smoking visitors are used to rules	Unclear due to the many exceptions	Unclear due to the many exceptions	Rules accepted<br>Smoking visitors react positive to rules<br>Most smokers they know smoke in kitchen or outdoors	Unclear due to the many exceptions
**Enforcement of HSRs**	Many exceptions and violations<br>Difficulties with communicating and enforcing rules (no assertiveness, no agency)<br>Rules and enforcement depend on mood	No exceptions or violations allowed<br>Communicates rules if necessary<br>Feels not in position to set stricter rules (no agency, as partner is the smoker)<br>Easy to maintain rules	Often exceptions allowed (e.g., smoking in living room when children are asleep)		Often exceptions allowed (e.g., when child is not at home)	Limited enforcement<br>Often exceptions allowed (e.g., indoor smoking in weekends, when children are asleep, bad weather)<br>Feels not in position to maintain rules or set stricter rules (no agency, as being a smoker too)

Respondent number and parental smoking status	R04 Non-smoker Single parent	R07 Non-smoker Partner: smoker	R02 Smoker Single parent	R03 Smoker Partner: non-smoker	R06 Smoker Partner: non-smoker	R13 Smoker Partner: smoker
**Smoking habits and HSRs**		Smoking in kitchen has become habit<br>Partner already used to smoking in kitchen	Difficult to change smoking habit toward smoking in kitchen		At first difficulties with changing smoking habit into smoking in kitchen<br>Smoking in kitchen has become habit by now	
**Intention to have SFH**	Intention for SFH	No intention for SFH<br>SFH would be the same as smoking cessation<br>Wants partner to quit smoking	Would like to have SFH<br>SFH not realistic as next step<br>Present HSRs already difficult enough<br>SFH more difficult than quitting smoking<br>Want to quit smoking for health reasons	Would like to have SFH<br>Tries to smoke outside in case of good weather<br>Prefers to quit smoking (above creating SFH) because of own health and health of children	Intention for SFH<br>SFH is logical next step<br>Tries to smoke outside in case of good weather	No intention for SFH<br>Smoking outdoors not acceptable as it interferes with enjoying life<br>Prefers partner to quit smoking<br>Want to continue smoking during special moments
**Motivation for SFH**	Smoke smells and is dirty		Own health and health of others<br>Smoke smells	Health<br>Fresh house	Own health and health of others<br>Smoke smells and is dirty<br>Better for environment<br>May lead to less smoking<br>Need for external motivator (like puppies)	
**Barriers to SFH**	No comfortable outdoor place to smoke<br>Balcony not acceptable (next to bedroom child)<br>SFH could spoil coziness of smoking indoors<br>No valid reasons for stricter HSRs	No outdoor space at all	Bad weather<br>Feeling unsafe outdoors in the evening<br>Smoking in kitchen already less comfortable than in living room<br>Outdoor smoking even more uncomfortable	Bad weather	Bad weather<br>Smoking in kitchen already less comfortable than in living room<br>Outdoor smoking even more uncomfortable (smoking for relaxation)	Bad weather<br>Addiction of partner

Respondent number and parental smoking status	R04 Non-smoker Single parent	R07 Non-smoker Partner: smoker	R02 Smoker Single parent	R03 Smoker Partner: non-smoker	R06 Smoker Partner: non-smoker	R13 Smoker Partner: smoker
**Facilitators for SFH**	Shelter for comfortable outdoor smoking place<br>Being more assertive to set and enforce rules<br>Non-smoking sign<br>Maybe support from well-known family supporter	Smoking cessation of partner	Shelter for a comfortable outdoor smoking place<br>Discipline and will power<br>Smoking cessation	Social support from partner<br>No need for other support	Maybe support from acquaintance (as buddy)	Comfortable outdoor smoking place (e.g., lightning, outdoor couch)<br>No need for support
**Role of children in HSRs, SFH or quitting smoking**	Should communicate and enforce rules among smoking friends<br>Determining a reward for obeying rules	Have important role (it concerns their future)<br>Could confront smokers (e.g., to create feelings of guilt, as to have impact)	Could confront parent with health consequences of smoking<br>Could support parent in quitting smoking<br>Should have mercy with parent because difficulty of quitting	Could confront parent with health consequences of smoking<br>Could tell parent that the house smells<br>Could advice parent to quit smoking<br>Could support parent in quitting smoking	Could confront parent with health consequences of smoking<br>Could tell parent that the house smells<br>Could ask parent to smoke outside<br>Giving young children such a role is not fair as smoker is responsible	Have no role because they don't know that parents smoke inside<br>Children do not confront parents with their smoking behavior<br>HSRs are primarily responsibility of parents/smokers
